# Real-time tracking of ionic nano-domains under shear flow

**DOI:** 10.1038/s41598-021-98137-y

**Published:** 2021-10-01

**Authors:** Clodomiro Cafolla, Kislon Voïtchovsky

**Affiliations:** Physics Department, Durham University, Durham, DH1 3LE UK

**Keywords:** Rheology, Surfaces, interfaces and thin films, Fluid dynamics, Scanning probe microscopy

## Abstract

The behaviour of ions at solid–liquid interfaces underpins countless phenomena, from the conduction of nervous impulses to charge transfer in solar cells. In most cases, ions do not operate as isolated entities, but in conjunction with neighbouring ions and the surrounding solution. In aqueous solutions, recent studies suggest the existence of group dynamics through water-mediated clusters but results allowing direct tracking of ionic domains with atomic precision are scarce. Here, we use high-speed atomic force microscopy to track the evolution of Rb^+^, K^+^, Na^+^ and Ca^2+^ nano-domains containing 20 to 120 ions adsorbed at the surface of mica in aqueous solution. The interface is exposed to a shear flow able to influence the lateral motion of single ions and clusters. The results show that, when in groups, metal ions tend to move with a relatively slow dynamics, as can be expected from a correlated group motion, with an average residence timescale of ~ 1–2 s for individual ions at a given atomic site. The average group velocity of the clusters depends on the ions’ charge density and can be explained by the ion’s hydration state. The lateral shear flow of the fluid is insufficient to desorb ions, but indirectly influences the diffusion dynamics by acting on ions in close vicinity to the surface. The results provide insights into the dynamics of ion clusters when adsorbed onto an immersed solid under shear flow.

## Introduction

Metal ions are ubiquitous in nature and present in most aqueous media where they tend to accumulate at the interface with immersed solids. They play a key role in a wide spectrum of interfacial phenomena and technological applications^1^ ranging from protein folding^2^ and cell membrane mechanics^3^ to electrochemistry and energy-related application^4,5^, tribology and lubrication^6,7^. Solids typically develop a surface potential when immersed in aqueous solutions, leading to the adsorption of counter-ions. Once adsorbed, metal ions can modulate the properties of the interface, acting directly through electrostatic effects^8,9^ and indirectly by modifying the interactions of the surface with the surrounding medium^7,10–12^. Examples span from biological enzymes^13^ to sliding surfaces^7^, nanotubes^8^ and graphene oxide membranes^14^.

The behaviour of ions at solid–liquid interfaces is often described using continuum models such as the Gouy-Chapman-Stern^15^ or the Dynamic Stern Layer^16^ models. While highly successful for describing macroscopic systems at equilibrium, these models can fall short at the nanoscale when atomistic details such as specific solvation effects and local defects or singularities become important^7,15,17–22^. For example, the specific organisation of ions into domains is common in biomedical and technological processes such as the effects of lithium on mitochondrial membranes^23,24^ and ion transport through nanofluidic channels^8,25^. Part of the problem comes from the difficulty in obtaining suitable measurements able to inform theoretical developments^26–29^. Tracking the organisation and dynamics of individual ions at interfaces is highly challenging^7,17,30–34^, especially when out of equilibrium or during electro-kinetic phenomena^35–37^. Computer simulations can capture ions’ femto- to nanosecond dynamics^38,39^, but with current capabilities, most investigations are limited both in number of atoms and in their full duration, rarely going beyond hundreds of nanoseconds^38,40^. Additionally, key effects, such as water molecules dissociation, pH and mobility^39,41^, are difficult to account for. Experimental approaches such as X-ray reflectometry^42–45^ and neutron scattering^46,47^ can probe the interface exchange dynamics, but they average over hundreds of thousands of ions and can be limited in their time resolution.

Atomic force microscopy (AFM) can image single ions adsorbed at various solid–liquid interfaces^7,17,30,33,48–50^. One of the main advantages of the technique is its ability to probe individual ions *in-situ* but with local contextual information about the interface over tens of nanometres at the point of measurement^7,30,48,50^. The relatively slow temporal resolution of standard AFM (typically 30–100 s per image) limits its use for obtaining dynamical information, but multiple improvements to the technique over the last decade have enabled the emergence of high-speed AFM (HS-AFM). HS-AFM operates similarly to standard AFM but with enhanced temporal resolution and can capture images at video rate, making it possible to track many molecular processes in real-time^51–53^.

In this study, we use HS-AFM to track the dynamics of ionic nano-domains adsorbed at the mica-water interface when subject to a lateral shear flow of the liquid. We achieve single-ion spatial resolution with ~ 2 s temporal resolution. While higher temporal resolution is technically possible, we aim to strike a compromise between retaining atomic resolution and improve temporal resolution in solution. Using chloride salts (NaCl, KCl, RbCl, and CaCl_2_ all at 5 mM concentration) dissolved into ultrapure water, we examine the impact of the cations’ charge density on the organisation and velocity of the adsorbed nano-domains under shear flow. Mica being a well-established model surface to study ion dynamics^7,30,45,54^ allows for a better interpretation of the results, highlighting the interplay between electrostatic interactions and solvation forces at the interface and the remarkably slow dynamics of the adsorbed ionic clusters.

## Results and discussion

**Figure 1:**
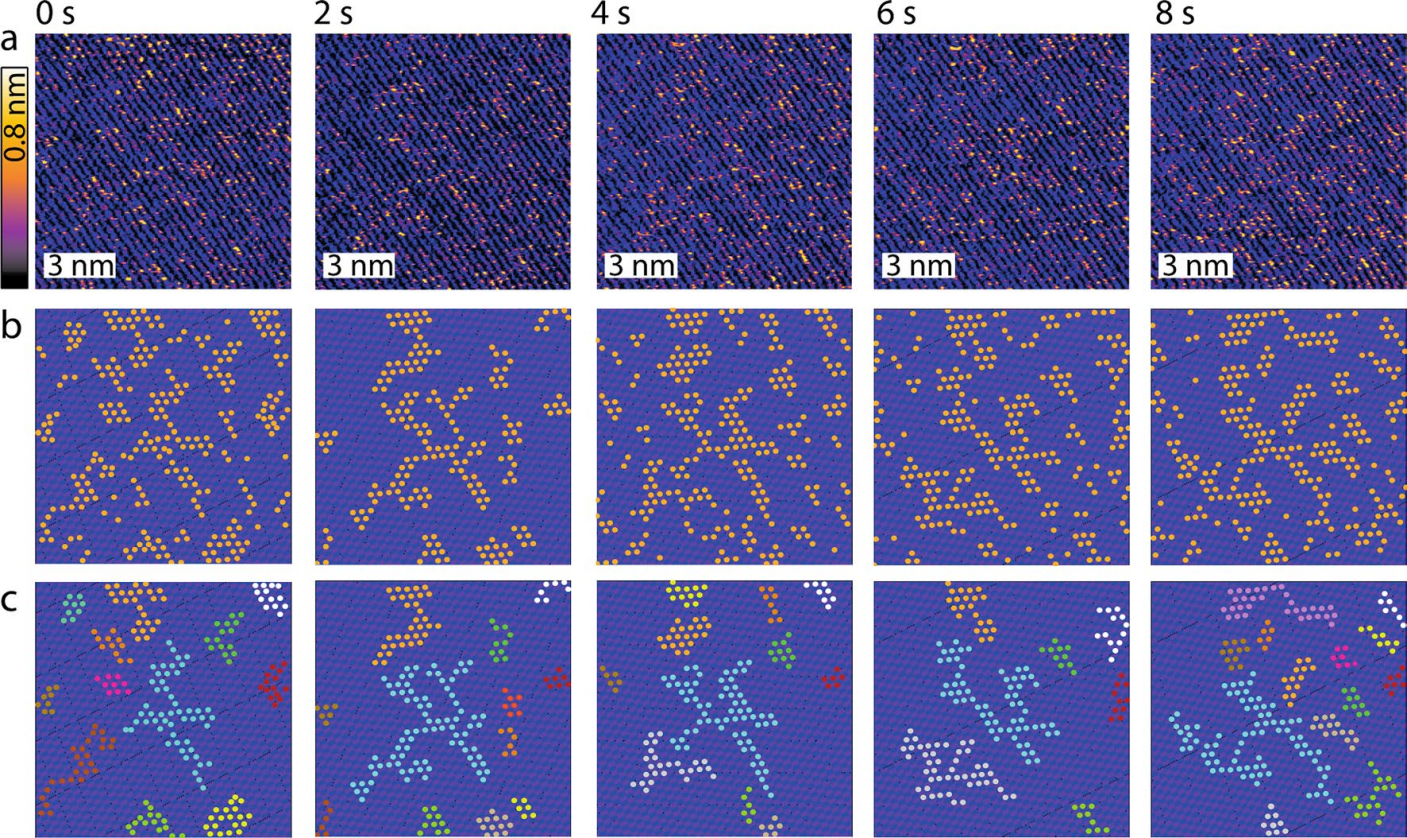
Example of time evolution for adsorbed Rb^+^ ions at the mica-water interface in the presence of a shear flow. (**a**) A time-lapse sequence shows consecutive high-resolution HS-AFM topographical images of Rb^+^ ions at the interface between mica and a 5 mM RbCl aqueous solution. Rb^+^ ions appear as bright orange-yellow protrusions standing taller than the mica surface (purple-black). Periodic rows and larger domains are clearly visible as well as singly adsorbed rubidium ions. (**b**) Representative image analysis highlighting Rb^+^ ions as orange markers (obtained by thresholding) and the idealised underlying lattice derived by inverse Fourier transform of the filtered power spectrum in each image of (**a**) (see ESI Section 3 for details on the procedure). (**c**) The algorithm automatically associates neighbouring ions (within distances < 0.52 nm) to the same cluster. Domains smaller than 5 ions are discarded here. The different clusters derived in each image are highlighted using different colours, keeping for each cluster the same colour over time. The cyan-coloured cluster offers a good example of temporal evolution. The scale bars in (**a**–**c**) represent 3 nm and the z-scale in (**a**) corresponds to 0.8 nm.

In this study, the HS-AFM is operated in amplitude modulation^7,30,55–57^ for the robustness of the operating mode at high speed^51,58^ and the demonstrated ability of the mode to achieve single ion resolution^7,17,30^. Practically, the oscillating tip explores the mica-water interface using relatively small oscillation amplitudes (< 1 nm), to detect local changes in the interfacial water organisation. Such changes can be induced by adsorbed ions or reflect local variations in the solid’s physical and chemical atomic structure. Hydrated metal ions located within the Stern layer typically appear as protrusions in the topography^7,17,59^ with an associated ion-specific phase shift in the cantilever vibration^7,49,60^. Here we focus on the topographic information to objectively determine the position of each ions using a thresholding approach. A typical time-lapse sequence of topographic images obtained at 2 s/frame and atomic-level resolution is shown in Fig. 1a, acquired in an aqueous solution of 5 mM RbCl. The ions appear as bright (orange-yellow) protrusions on the darker (purple-black) mica lattice. Most ions do not appear as isolated protrusions, but rather as part of a larger cluster due to water-mediated attractive correlations effects^30^. The resulting mesoscale patterns evolve over time (Fig. 1a), confirming the presence of mobile entities^17^. These patterns depend on the nature of each adsorbed cation species, modulated by both the local electrostatics and the interplay between the hydration structures of the mica and the ions^17,30,42,44^. The videos V1–4 in Section 1 of the ESI show the evolution of four different types of ions: Rb^+^, K^+^, Na^+^, Ca^+2+^. The contribution from the chloride anions in the HS-AFM images is negligible at the salt concentrations investigated here, due to the strongly negative surface charge of the mica (approximately − 0.34 C/m^2^) at the experimental pH^61,62^ (see “Materials and methods”). Cation adsorption to the negatively charged silicon nitride AFM probe may occur, but it is unlikely to influence the observed cluster dynamics considering the tip sharpness and the relatively low charge density (typically < 100 mC/m^2^) at the pH values used here^63^. Multiple independent locations were tested confirming the reproducibility of the results for the different ions investigated. To objectively analyse the spatial and temporal evolution of the cation clusters, for each frame the locations of the adsorbed ions were identified using a custom-made semi-automatic algorithm based on height-thresholding (see ESI Section 2). The thresholding strategy is not perfect, being sensitive to local changes in the interactions between the scanning tip and the interface. Such changes can occur during imaging, for example due to local molecular fluctuations or occasional atomic changes in the structure of the tip^59^. These issues are overcome by a statistical approach where the same threshold is used throughout all the images of a given experimental series. While individual false detection can occasionally occur, the statistical nature of the analysis ensures that the findings identify robust trends, at least for the relatively low ionic concentrations used in this study.

**Figure 2:**
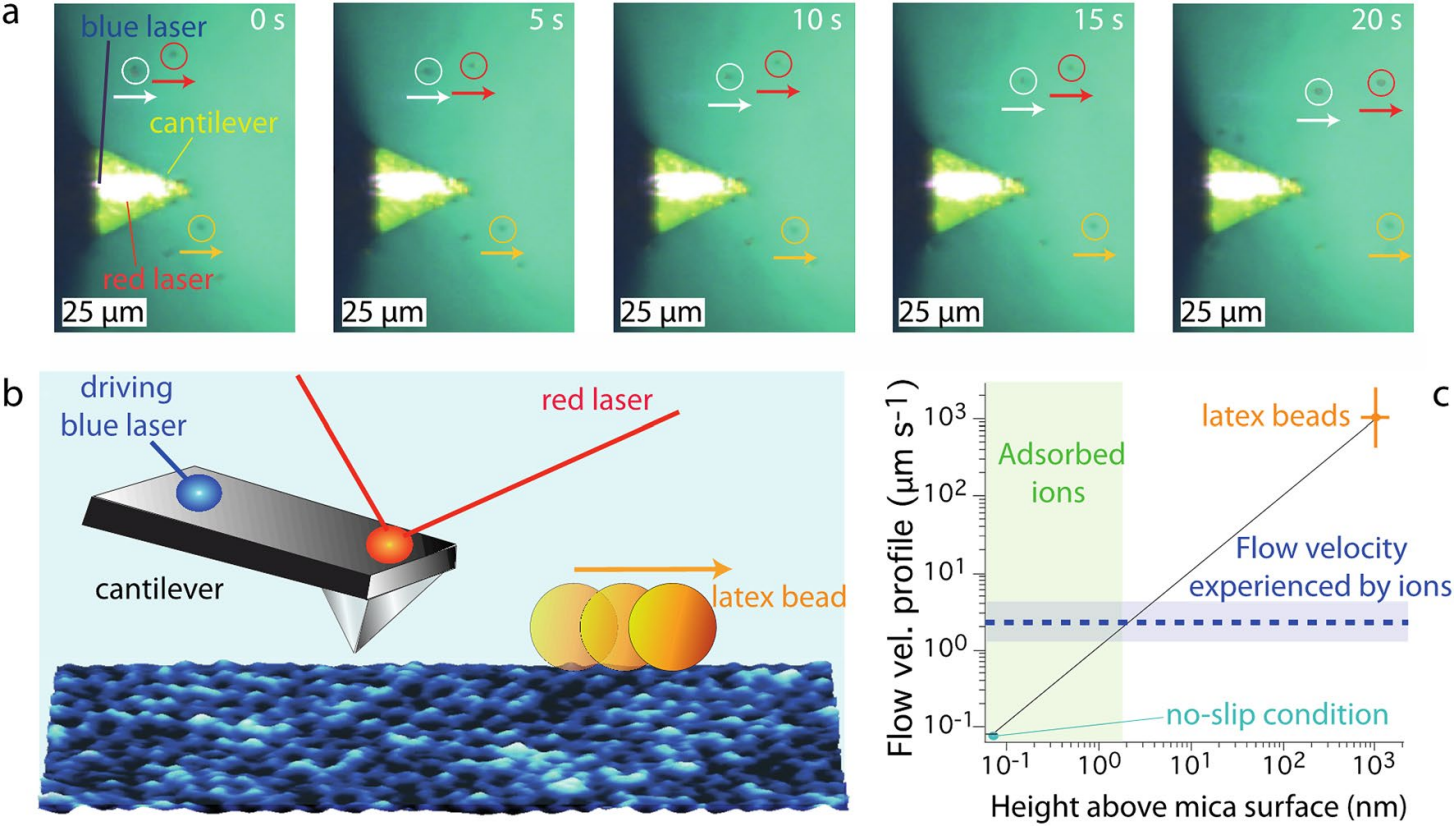
Shear flow analysis. (**a**) Representative time-lapse optical microscopy images of latex beads moving away from the AFM cantilever. The latex beads are highlighted by yellow, white and red circles. (**b**) Schematics highlighting the key events at the water-mica interface. The cantilever is photothermally excited by a blue laser focused near its base. The red laser deflection is used to track the cantilever motion. The periodic motion of the cantilever driven by the blue laser combined with the heat transferred to the fluid by the blue laser itself generate a thermal gradient responsible for the shear flow the latex beads experience. The orientation of the shear flow with respect to the cantilever is the same as in Fig. 1. (**c**) Assuming a no-slip boundary condition for the water-mica interface^7,65^, the fluid velocity profile for the adsorbed ions can be inferred from the measured latex bead velocity profile (~ 3 μm/s). The motion of the latex beads was calculated assuming a distance from the surface equal to the radius of the particles (focus point of the microscope). The value and associated error in (**c**) represent the average and standard error from three independent experiments performed using different cantilevers, solutions, and mica substrates. All the experiments were performed at thermal equilibrium (25.0 ± 0.1 °C), based on a constant heating/cooling rate of the AFM temperature control system. Within each experiment, at least three sets of data tracking the dynamics of the latex beads were captured and analysed. The results confirmed reproducibility and consistency of the shear flow with minimal fluctuations when operating at thermal equilibrium.

**Figure 3:**
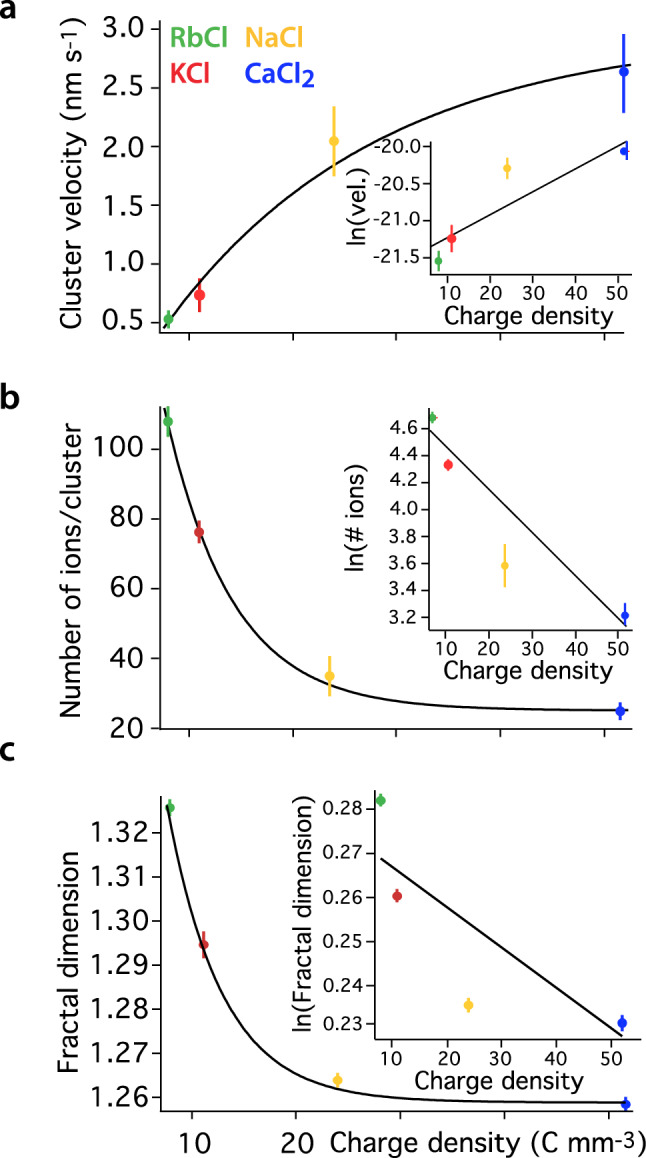
Dynamics of cationinc clusters at the interface. (**a**) The clusters’ mobility is calculated considering the average displacement of the cluster's centre of gravity between consecutive frames. The average cluster velocity increases with *ρ* and can be modelled with an exponential behaviour. This is consistent with a hydration-dominated process whereby thermal fluctuations govern the adsorption/desorption of ions from the mica surface. More strongly hydrated ions are moved more easily due to a weaker adsorption when in outer sphere configuration^45^ (see text). (**b**) The average number of ions per cluster decays exponentially with *ρ*, as does the average size of the cluster (see Fig. S4 within ESI Section 6). This suggests higher *ρ* to disfavour attractive water-mediated correlation interactions between ions^30^. Consistently, the fractal dimension (**c**) of clusters decays with *ρ*, reflecting the ability of ions with smaller *ρ* to create more complex cluster shapes due to stronger water-mediated attractive interactions (as visible in Fig. 1). The number of ions per cluster, the domain size and the fractal dimension are averaged over all the frames. ESI Section 7 provides further details on estimating fractal dimension. The insets in (**a**–**c**) suggest an exponential dependence on *ρ*.

Figure 1b illustrates the thresholding analysis performed on the images presented in Fig. 1a. Once an ion is identified, its immediate surrounding is analysed for possible neighbours. Two ions at a distance equal or smaller than a mica lattice constant (i.e. < 0.52 nm)^7,64^ are ascribed to the same cluster (Fig. 1c). Domains with fewer than 5 ions were not considered here because they rarely survive more than 2 frames, making meaningful tracking and analysis difficult. As clear from Fig. 1, the clusters tend to all move in the same direction, here from left to right. Aside from local thermal fluctuations, the general direction of motion coincides with a shear flow of water parallel to the mica surface. The flow is due to the photothermal actuation of the imaging cantilever (blue laser) locally dissipating several milliwatts of power during the experiment. As a result, and due to the particular geometry of the setup, a lateral flow moving away from the photothermal excitation point can be observed, confirmed by micron-size tracker beads dissolved in the solution and directly visible optically (Fig. 2a). The use of latex tracker beads of known size (2 μm) allows for direct quantification of the lateral flow in the AFM imaging region. This is done using optical tracking with the same objective that serves to focus the laser on the back of AFM cantilever. By selecting beads with a radius comparable to the height of the AFM tip, optical tracking of the beads allows for direct *in-situ* quantification of the lateral flow at the point of focus, here the back for the cantilever which is at a known distance from the mica surface when imaging (Fig. 2b). Additionally, the use of a thermally induced flow is considerably simpler to implement and less noisy than a pressure induced flow, for example using a liquid exchange setup. The latter often renders simultaneous high-resolution imaging challenging. Here, we estimate the flow velocity experienced by the ions at the interface by relying on the well-established no-slip boundary condition for mica^65^, and inferring a linear flow profile from the point of measurement (Fig. 2c). The ions being typically ~ 0.3 nm above the average height of the bare mica surface (Fig. 1a), the lateral water velocity they experience is in the order of a few nm/s (Fig. 3a). A crude estimate of the resulting force exerted on a single ion can be inferred from Stokes law yielding values in the order of *F*_*flow*_ ~ 10^–21^ N (see ESI Section 4). While such an estimate is unlikely to faithfully account for the molecular interactions on the scale of a single ion, it provides an order of magnitude for the associated work: over a distance of a single lattice site, the resulting energy is several orders of magnitude smaller than the thermal energy or the electrostatic energy binding ions to the mica surface (see also ESI Section 5). This indicates that the flow does not influence bound ions but may only play a role when ions are detached from the surface through a global, average bias of the thermal diffusion.

The estimate of the shear force experienced by the ions relies on several assumptions when interpreting the bead tracking data. First, the flow is assumed constant and regular. This was verified by repeating each experiment multiple times and ensuring thermal equilibrium. The assumption of a linear flow profile is to some extent arbitrary, but it provides a simple framework to estimate the magnitude of the shear flow experienced by the ions, based on the beads’ velocity. This assumption is unlikely to hold for all distances from the surface. Additionally, in this framework, the beads experience a differential flow with possible rolling and measurements taken at a set distance from the surface cannot capture this level of detail. However, these simplifications are unlikely to change the order of magnitude of the estimated shear flow experienced by the ions. The conclusion that the shear flow is insufficient to directly remove ions from the surface stands.

Before analysing the HS-AFM data in details, it is crucial to disentangle the genuine dynamics of the observed ions from possible imaging drift, a common issue in scanning probe microscopies^66–68^. Here, this was done by analysing the apparent distortion of the mica lattice with respect to the expected perfectly hexagonal mica lattice. Since the experiments were conducted with a thermally equilibrated AFM, it is reasonable to assume any drift to be constant over time, hence resulting in a small and linear distortion of the apparent lattice in AFM images. Practically, the drift analysis can be conducted over each AFM image based on the image power spectrum (Fourier transform) so as to precisely identify the average apparent lattice. The power spectrum is then compared with that of a perfect lattice based on the same reciprocal first lattice point. This allows estimating the drift over the whole image (see ESI Section 3 for details and an example of the procedure). From this analysis, a typical drift rate of 0.1 nm/s (0.2 nm/image) could be identified. While not negligible, it is significantly smaller than the smallest possible displacement of one lattice parameter for a moving ion between two consecutive images, indicating that results from the ion tracking can be safely ascribed to ion dynamics at the interface and not to imaging drift.

The analysis resulting from the tracking of ionic clusters over multiple images is presented in Fig. 3. The analysis focuses on longer-lived domains identified as those surviving for more than 6 s (at least 4 frames) to limit the uncertainty inherent to transitions from single to multiple domains or reciprocally, and exclude clusters with insufficient temporal information. The dynamics of the clusters clearly depends on the ion’s charge density, *ρ*, (Fig. 3a) with an average cluster velocity increasing exponentially with *ρ* (inset).

Considering the strongly negative surface charge of mica^61,62^, the fact that ions with lower charge density appear less mobile may seem counter intuitive. However, to get a full picture of the physics at play, it is necessary to consider the hydration of the ions and the mica, and the fact that it is not the mobility of isolated ions that is considered here, but that of ions in clusters.

The adsorption and desorption dynamics of ions at the interface depends on the partial disruption of their hydration shells^45^. During desorption, ions change their configuration from inner-sphere (IS) to outer-sphere (OS) complexes (see Fig. S5 in ESI for a pictorial representation of the two configurations). The former corresponds to a partially solvated state with the mica surface completing the solvation structure and direct electrostatic interactions between the ion and the surface, whereas OS complexes are fully hydrated and thus located further away from the mica surface^42^. The hydration/de-hydration energies involved are considerable and the transition between configurations can take seconds and follow several possible pathways^45^, with the exchange favoured by thermal fluctuations. The ions in OS configuration being located further away from the mica surface, they are more visible in the AFM images and inevitably the main ions taken into account by the thresholding analysis. As a result, more densely charged cations appear more mobile because they sit further from the mica surface than cations with lower charge density when in OS configuration. XXR studies have showed that when in OS, the more strongly hydrated cations such as Na^+^ have a lower affinity for the mica surface than the less strongly hydrated cations (K^+^ and Rb^+^). Na^+^ ions tend to adsorb dominantly as OS complexes and hence tend to stay at a larger distance from the mica surface compared to K^+^ and Rb^+^ which adsorb mainly as IS complexes^42,54^ (see also ESI Sections 2 and 8). The smaller the value of *ρ*, the greater the fraction of cations adopting an IS configuration^42,45^. Divalent ions, however, have an effective higher size due to their tightly bound first hydration shell which decreases the adsorption available area^69,70^.

Once released from the surface, the ions can rebind or move to nearby locations through thermal diffusion, here globally biased by the shear flow. The electrostatic and mechanical interactions induced by the scanning probe (~ 0.5–5 *k*_*B*_*T*)^30^ may also play a role by increasing the effectively local thermal energy, but not sufficiently for the tip to appreciably alter the ions’ dynamics in the clusters investigated. Shear experiments imposing a strong tip confinement over single adsorbed ions revealed significant entropic costs associated with the dehydration of the confined ions^7^ and impacting the resulting interface dynamics. Here, the scanning probe operates in soft tapping and avoids strong confinement thereby limiting perturbation to the ions’ hydration shells. Smaller clusters (not considered in this work) may be destabilised by the scanning tip due to the limited influence of cohesive water-mediated correlation effects. Individual ions can still undergo desorption from the surface or adsorption from the bulk. This intrinsically stochastic process reflects the entropic nature of the hydration effects and renders smaller domains more prone to fluctuations between consecutive images, for example, in Fig. 1b,c at 2 s where an apparent depletion of the smaller domains occur.

When observing ions clusters (Fig. 3a), the group dynamics remains consistent with a thermally activated process whereby thermal fluctuations and perturbations from the scanning tip allow ions to desorb from the mica binding sites and move to adjacent sites. The fact that ions tend to move as a cluster can be due to either electrostatic correlation effects or to water-induced effects. Estimates of the magnitude of the electrostatic interactions at play indicate that they are insufficient to robustly stabilise ionic domains over the time scale observed here. This is all the more obvious considering the fact that divalent cations form smaller, less stable domains (see ESI Section 5). Instead, the attractive correlation interactions are best explained by previously reported hydration effects^30^ whereby neighbouring ions can increase the entropy of the system by sharing hydration water molecules, thereby releasing otherwise trapped water into the bulk. These interactions enables clusters to move as a unit over the measurement timescale with limited restructuring^17,30,45^. Water-induced correlations depend on the specific hydration characteristics of a given ion type and are much more effective for smaller charge density. The predominant hydration state (IS or OS), itself directly controlled by *ρ*, also influences the hydration effects. Rb^+^ and K^+^ tend to have one single hydration state and remain at their site once adsorbed, thus favouring the formation of a stable hydration structure^30,42^. In other words, ions with a smaller charge density experience correlations strong enough to stabilise clusters several nanometres in diameter (Fig. 3b,c). Water-mediated interactions are much less effective in arranging Na^+^ and Ca^2+^ ions in a correlated structure near the surface. This is due to the multiple solvation states and relatively high mobility of Na^+^ ions ^30,42^, and to relatively low adsorbed density of divalent ions because of their double charge^69,70^. Correlation effects, as modulated by *ρ*, also influence the changes in size the clusters may experience over time. More mobile domains tend to experience increased restructuring with Rb^+^ and K^+^ clusters appearing more stable over time (see ESI Section 9 for a more detailed analysis).

Within this framework, the dynamics of the domains is highly sensitive to *ρ*. Ions with a smaller charge density tend to sit closer to the surface and form strongly correlated domains resulting in slower overall dynamics. The exponential dependence on *ρ* supports the idea of a thermally activated motion where thermal energy balances electrostatic and hydration interactions (see also ESI Section 6). As a result, adsorbed Rb^+^ tend to form relatively large, complex and slowly moving, ions compared to the more densely charged Na^+^ and Ca^2+^. The clusters made of ions with lower *ρ* also appear more complex and exhibit a higher fractal number. The existence of fractal-like ionic clusters is reminiscent of those formed on a larger scale by aggregating colloidal particles, depending on the shape of the inter-colloid potential^71^. Fractal structures are favoured by barrierless always attractive interactions whereas the addition of an energy barrier (repulsive interaction at longer range) tends to decrease fractal dimension of the colloidal aggregates. This is because the energy barrier allows particles to rearrange and move closer together before getting ‘stuck’ by the attractive interaction at shorter range. In this analogy, water-induced correlation effects (water sharing) play the role of attractive interactions for the ions forming clusters at the interface with mica, whereas the dehydration energy associated with the removal of hydration water molecules between approaching ions provides the energy barrier if uncompensated. At shorter distances electrostatics would become dominant, but the mica lattice imposes the inter-ion distance. In addition, hydration water help shield the ion's electric field^69,72^. Ions with larger *ρ* have naturally more hydration water molecules, including tightly bound molecules that may not be able to rearrange favourably when ions sit on adjacent mica sites. While helpful to explain the experimental observations, this analogy remains partly speculative with further work needed to provide a definitive explanation of the underpinning molecular mechanisms.

## Conclusions

This study uses HS-AFM with atomic-level resolution to track the dynamics of single metal ions at the water-mica interface. The combination of sub-nanometre lateral resolution and second time resolution reveals the organisation and evolution of single ions into clusters when subject to a lateral shear flow of the liquid. The dynamics of the ions depends on their charge density with higher charge density leading to faster dynamics and reduced group effects. This can be rationalised by considering the structure and dynamics of the ions’ hydration shell, either in solution or adsorbed at the surface of mica. Ions with lower charge density and smaller hydration shells are more stably adsorbed in inner sphere coordination. This, combined with relatively strong water-mediated attractive interactions between ions, leads to the formation of larger and more convoluted clusters which tend to move relatively slowly. When increasing the charge density, metal cations tend to preferentially adsorb further away from the mica surface as fully hydrated complexes. The greater charge density also limits the effects of attractive correlations and promote the formation of smaller, more mobile domains. The effect of the water shear flow on the adsorption/desorption dynamics of the ions is negligible. However, once detached from the mica surface, the shear flow influences the diffusion direction of the ions until the next adsorption. This effect is amplified by the fact that the ions tend to move as a cluster.

The results provide some quantitative insights into the relationship between single ion properties and group dynamics at the solid–liquid interface in the presence of a microscale shear flow, with potential technological applications from manufacturing biomedical devices to enhancing the performance of aqueous ion-batteries.

## Materials and methods

High-quality V1 muscovite mica disc were purchased from SPI Supplies, West Chester, PA, USA. Mica discs were glued to a steel plate. The aqueous solutions were prepared in ultrapure water (Water AnalaR NORMAPUR, VWR International Ltd, Leicestershire, UK) with 99.9% pure salts (Sigma-Aldrich, St Louis, MO, USA). No buffering agent was used to avoid interfering with the measurements^7,73^. pH stability (~ 6.0 pH) was tested immediately before and after conducting experiments with variations smaller than 0.1, well above mica’s isoelectric point^74^. Green fluorescent latex beads (average particle size 2.0 μm) were purchased from Sigma-Aldrich, and diluted to 0.5% v/v using ultra-pure water. Extensive cleaning procedures ensured the reliability of the results, and the negligible final concentration of K^+^ ions initially present at the mica surface (see ESI Section 10).

The AFM experiments were conducted at room temperature using a VRS Cypher (Asylum Research, Oxford Instruments, Santa Barbara, CA, USA). Before the experiments, the mica disc was freshly cleaved. Both the mica disc and the cantilever tip were thoroughly washed with pure water (20 times with 100 μl) and then with the solution of interest (40 times with 100 μl). This ensured that only the metal ions of interest were present on the mica surface (see also ESI Section 8). Thorough cleaning procedures were implemented so as to avoid any possible sources of contamination^7,75^ (see also ESI Section 10).

During the measurements, the cantilever and the sample were fully immersed in the aqueous ionic solution of interest. The AFM probes were Arrow UHF silicon nitride cantilevers (Nanoworld, Switzerland). The thermal spectrum of the cantilever was used to perform the flexural calibration of the cantilevers^76^. The probes were found to have a flexural spring constant in the range 1.0–4.0 N/m and a resonance frequency of ~ 400 kHz in water. The values are in agreement with the literature^20,77^. The cantilever oscillation was photo-thermally driven so as to ensure greater stability. The AFM was operated in amplitude-modulation^19,55,75,78–80^ for imaging. The oscillation amplitude is kept constant during imaging by a feedback loop. The topography is reconstructed from the feedback corrections. The phase lag between the driving oscillation and the cantilever oscillation varies freely and carries information on the energy interactions between the cantilever tip and the interface^19,55,75,78–80^.

The latex beads motion experiments were performed depositing 100 ul of diluted particles on a freshly cleaved mica disc mounted within the AFM chamber. Particles motion was studied using the optical microscope integrated into the AFM. This was done while reproducing the experimental conditions of the AFM measurements, thus optically driving the Arrow UHF cantilever at a frequency close to its resonance, and with the cantilever fully immersed in the solution during the measurements.

Data analysis was conducted using the ImageJ/Fiji free software^81^ (latex particle tracking), and homemade routines developed in Igor Pro (Wavemetrics, Lake Oswego, OR, USA) and Python (AFM data analysis).

## Supplementary Information

Supplementary Information 1.

Supplementary Video S1.

Supplementary Video S2.

Supplementary Video S3.

Supplementary Video S4.

## Data Availability

The data is available upon reasonable request.
