# Supplementary material for: Real-time tracking of ionic nano-domains under shear flow
Source: Sci Rep. 2021 Oct 1;11:19540. doi: 10.1038/s41598-021-98137-y (PMC8486851; doi:10.1038/s41598-021-98137-y)
Supplement: Supplementary file 1 — Supplementary Information 1. [file 41598_2021_98137_MOESM1_ESM.docx]

**Supplementary Information**

**Real-time tracking of ionic nano-domains under shear flow**

Clodomiro Cafolla and Kislon Voïtchovsky

Physics Department, Durham University

**Index of contents**

1. Representative results: time evolution of ion clusters (videos 1, 2, 3, 4)
2. Extended image analysis (Fig. S1)
3. Assessing the imaging drift from lattice distortion (Fig. S2)
4. Estimation of the drag force experienced by ions due to the lateral shear flow
5. Electrostatic energy calculations (Fig. S3)
6. Ion clusters size as a function of charge density (Fig. S4)
7. Fractal dimension analysis
8. Pictorial representation of inner-sphere and outer-sphere complexes (Fig. S5)
9. Stability over time of ionic domains (Fig. S6)
10. Cleaning procedure and extended methods
11. Supplementary References

**1. Representative results: time evolution of ion clusters**

The supplementary videos V1-V4 show representative data sets tracking the interfacial dynamics of the adsorbed ions in RbCl, KCl, NaCl and CaCl_2_ 5 mM acqueous solutions.

**Video 1.** Example of time evolution of Rb^+^ ions adsorbed to the mica-water interface. A time-lapse sequence shows consecutive high-resolution HS-AFM topographical images of Rb^+^ ions at the interface between mica and a 5 mM RbCl aqueous solution. On the left the images are shown as acquired, and on the right the identified ions are highlighted with yellow markers (see ESI section 2 below for further details on ions identification).

**Video 2.** Example of time evolution of K^+^ ions adsorbed to the mica-water interface. A time-lapse sequence shows consecutive high-resolution HS-AFM topographical images of K^+^ ions at the interface between mica and a 5 mM KCl aqueous solution. On the left the images are shown as acquired, and on the right the identified ions are highlighted with yellow markers (see ESI section 2 below for further details on ions identification).

**Video 3.** Example of time evolution of Na^+^ ions adsorbed to the mica-water interface. A time-lapse sequence shows consecutive high-resolution HS-AFM topographical images of Na^+^ ions at the interface between mica and a 5 mM NaCl aqueous solution. On the left the images are shown as acquired, and on the right the identified ions are highlighted with yellow markers (see ESI section 2 below for further details on ions identification).

**Video 4.** Example of time evolution of Ca^2+^ ions adsorbed to the mica-water interface. A time-lapse sequence shows consecutive high-resolution HS-AFM topographical images of Ca^2+^ ions at the interface between mica and a 5 mM CaCl_2_ aqueous solution. On the left the images are shown as acquired, and on the right the identified ions are highlighted with yellow markers (see ESI section 2 below for further details on ions identification).

The z-axis colour scale of the original videos is modified in Fig. 1 and Fig. S1 so as to improve the contrast between the lattice and the ions. This does not affect the results as the image analysis is automatically performed with an algorithm which operates independently from the colour scale. The algorithm is described in ESI section 2 Extended image analysis.

**2. Extended image analysis**

AFM images were automatically processed by a custom-made algorithm to automatically identify the ions. Representative results are shown in Fig. S1.

We firstly reconstructed the lattice of each image using its power spectrum (see also the following ESI section). Image noise was reduced using a square averaging filter of 256 pixels, corresponding to the number of pixels per image. Pixels were identified as corresponding to adsorbed ions if their height was, at least, 2 standard deviations greater than the mean height of all the pixels^1^. One of the main advantages of such an approach is that it does not require manually tuning the threshold for differently adsorbed ions thus opening the door for subjective decisions that would prevent comparison between images. Ions with a larger charge density tend to sit further away from the surface given the preferential adsorption as outer-sphere (OS) rather than inner-sphere (IS) complexes, typically ~ 0.25 nm further away, the size of a water molecule^2^ (see also Fig. S5). For both OS and IS complexes, adsorbed ions are automatically identified when the threshold condition is met, that is, when their height is greater than, at least, 2 standard deviations above the mean height of all the pixels. This criterium has shown to provide the best results in the present experimental conditions.

The procedure is illustrated in Fig. S1c-f showing the height profile corresponding to the sections (dashed white lines) taken on images Fig. S1a-b.

Here, the height is taken to be the root-mean squared roughness, *R_q_*^3^:

$R_{q}=\sqrt{\frac{1}{KL} \sum_{i=0}^{K-1} \sum_{j=0}^{L-1} \left| z\left( x_{i},y_{j} \right) \right|^{2}}$ (S1)

where *K* and *L* are the total number of pixels per row and column, respectively. Here, *K*=*L*=256. *z* is the height at the point *(x_i_,y_j_)* which is summed all over the *i* rows and *j* columns^1,3^.

Neighbouring ions within 0.52 nm from each other are automatically assigned to the same cluster which is visually identified by a colour and a number. The use of the numbers helps the visual inspection of the domains particularly in the case of frames containing a large number of domains with similar colours. In Fig. 1c, we omitted the numbers associated to the domains in order to improve clarity of the Figure.

It is worth mentioning that this approach becomes less accurate at higher salt concentration due to the higher surface coverage. Using an average threshold is likely to underestimate the number of ions adsorbed due to the threshold increasing with coverage. This was not an issue here given the relatively low ionic concentration, and the visual check of the thresholding result. However, previous studies on the same system have shown that threshold-based detection can become problematic above ~10 mM for simple metal ions on mica and other methods should be preferred^4^.

**Fig. S1.** Representative analysis of a single HS-AFM image from a video. (**a-b**) The video frames are processed line by line identifying the ions using a height threshold criterion. (**c-d**) The features taller than the average plus 2 standard deviations are identified as ions. (**e-f**) the ions are highlighted by orange marks.

***Tracking the evolution of clusters***

Clusters are systematically identified by the analysis software using the condition of neighbouring ions being closer than 0.52 nm (mica lattice spacing) for them to be allocated to the same domain. This occurs independently for every image. Second, the software compares clusters between consecutive images and identifies evolving clusters based on their positional overlap between consecutive images. Considering a particular cluster in a given image several situations occur:

1. A single cluster with some positional overlap (or a single site away) existed in the previous image. The new cluster is then identified as that in the previous image.
2. Several new clusters (based on the 0.52 nm criterion) appear where previously a single cluster was present. Then, the largest new cluster is given the identity of the previous cluster while the other clusters are treated as new. This typically occurs when a large cluster splits.
3. No cluster with positional overlap existed in the previous image; the cluster is treated as new.
4. Several clusters in the previous image have positional overlap with the cluster. The largest is identified with the cluster while the smaller are considered extinct.

**3. Assessing the imaging drift from lattice distortion**

Drift can be due to a number of different factors from thermal instabilities to incorrect calibration of the piezo constants of the scanning actuators^5^. Virtually all scanning probe microscopy measurements contain some level of spatial drift resulting in image distortions, such as shear, expansion or contraction of the crystal structure with respect to the true lattice^6,7^.

Different algorithms have been developed to measure the drift distortions^5,6,8^. 2D Fast Fourier Transform (FFT) analysis has emerged as a powerful technique allowing quantitative evaluation of the lattice drift by examining the distortion of the crystal structure^3^. The analysis is based on comparing the power spectrum of the acquired images with the ideal reciprocal lattice, that is, in the absence of any drift distortions^9–11^.

Representative results are shown in Fig. S2. The power spectra of the images are semi-automatically processed and the maxima identified fitting the region of interest with a 2D Gaussian function (Fig. S2). The reciprocal lattice (RL) points are compared with those of an ideal reciprocal crystal structure reconstructed using one of the RL points as the reference (Fig. S2a). As shown in Fig. S2b, the RL points are shifted by 5-10% with respect to the ideal reciprocal lattice. Given a scanning frequency per image ~ 0.5 Hz, this results in a drift ~ 0.1 nm/s. The inverse FFT images confirm the drift distortions of the mica lattice (Fig. S2d) in comparison with the ideal crystal structure (Fig. S2c). Even if not negligible, the drift is, at least, 5 times smaller than the observed mobility of the ions, and even one order of magnitude smaller in the case of divalent ions.


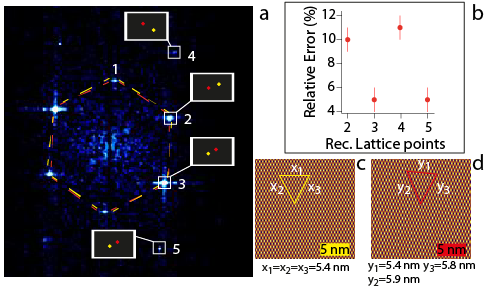


**Fig. S2**. Representative images of the FFT analysis to quantify the lattice drift. (**a**) The first 5 maxima in the power spectrum are identified using a 2D gaussian function. Images being recorded along the [100] axis of symmetry, maxima 1, 2 and 3 correspond to the k-vectors for the 0.90 nm lattice parameter, whereas maxima 4 and 5 to the reciprocal vectors for the 0.52 nm unit cell edge^12^. Maximum 1 is used as a reference point to reconstruct the position of the other maxima for an ideal reciprocal lattice. The zoom-in images in (**a**) show the locations of the observed (red) and ideal points (yellow) in reciprocal space. Dashed lines among the maxima help highlight the drift distortion of the observed reciprocal lattice (red) with respect to an ideal one (yellow). (**b**) The locations of the observed and ideal reciprocal lattice points are quantitatively compared. This is done considering the distance between each observed (and ideal) maximum from the reference maximum 1. The discrepancy is at most ~ 10% leading, in real space, to a drift ~ 0.1 nm/s. (**c**) and (**d**) show the inverse FFT images of the observed (yellow) and ideal (red) reciprocal lattice, respectively. Here, the drift distortion is highlighted comparing the triangles in (**c**) and (**d**). The two triangles contain the same number of lattice points, and the regular polygon in (**c**) is clearly distorted in (**d**). This results in each lattice site being shifted by ~ 0.2 nm. Given the scanning frequency per image ~ 0.5 Hz, this further confirms the drift ~ 0.1 nm/s. x_1_, x_2_ and x_3_ are the side lengths of the triangle in (**c**), whereas y_1_, y_2_ and y_3_ the side lengths of the triangle in (**d**).

**4. Estimation of the drag force experienced by ions due to the lateral shear flow**

The motion of the photothermally excited cantilever and the heat transfer of the blue laser to the fluid result in a thermal gradient as highlighted in Fig. 2. The thermal gradient produces a shear flow associated to a drag force, *F_flow_*_._ The magnitude of the drag force on a sphere of radius *R* can be approximated using Stokes law:

$$F_{flow}=6 \pi\eta vR, (S2)$$

where *η* is the fluid viscosity (here, water at 25 °C, 890 μPa s ^4,13^) and *v* is the lateral shear flow velocity, and *R* taken as the radius of the hydrated ion^14^. This yields *F_flow_* ~ 5×10^-21^ N and the associated work done on an ion moving between two adjacent lattice sites is ~ 2×10^-30^ J, or ~ 6×10^-10^ k_B_T. While this is a coarse estimate based on continuum assumptions that may not be valid here, it shows that the drag energy is significantly smaller than any thermal instabilities and electrostatic or correlation interactions (see following ESI section 5). This demonstrates that the shear flow is not responsible for the desorption of the ions, but can contribute towards influencing their overall motion once desorbed from the mica lattice sites.

**5. Electrostatic energy calculations**

The ionic nano-domains, experimentally observed, may be stabilised either by direct electrostatic interactions between the adsorbed ions or by water-mediated hydration effects^15^. Correlative electrostatic interactions are particularly important in systems with high charge density, for example in the case of multivalent ions^16^ on DNA^17^.

Given the relatively low charge density of the metal ions explored here, direct electrostatic interactions are unlikely to play a key role, but a more quantitative conclusion can be derived by estimating the different contributions to the overall electrostatic energy of interacting cations. Since we are after an estimate, we assumed the adsorbed ions to be single point charges, the mica surface to be a lattice of static negatively charged binding sites and the water molecules to be a continuum medium. While these simplifications prevent the model from fully capturing the complex interplay of electrostatic interactions, the model can still offer an order of magnitude for the possible electrostatic contribution to the correlation interactions within ionic domains.

Following the same analysis as presented in reference^15^, we start modelling the electrostatic interactions between a cation and the negatively charged mica. Within this model, we only consider the interactions between the cation and its binding site, and ignore the rest of the surface. The approximation is justified by the fact that the surrounding binding sites are occupied by hydronium ions that can interact more strongly and sit closer to the mica surface than metal ions^4,15^. Thus, the electrostatic energy of a single ion with the mica is^18^:

$E_{el}=-\frac{l_{B}}{d_{1}} k_{B}T, ($S3)

where *l_B_* is the Bjerrun length, *k_B_* the Boltzmann constant, *T* the temperature and *d_1_* the distance between the metal cations and the mica surface oxygen atoms. *l_B_* is given by^18^:

$l_{B}=\frac{{z^{2}e}^{2}}{4 \pi\epsilon_{0}\epsilon_{r}k_{B}T} , ($S4)

where *z* is the charge of the interacting ions expressed in the elementary charge *e*, *ε_0_* the vacuum permittivity and *ε_r_* the dielectric constant of the medium surrounding the cations. Here, *ε_r_* is taken to be the average of the dielectric constant of mica (~7 ^19–21^) and of water (~80 ^22^). Thus at 298 K, *l_B_* is ~ 1.3 nm and ~ 5.1 nm for monovalent and divalent ions, respectively. An anomalous low dielectric constant for water has been reported in the case of strong nanoconfinement with *ε_r_* reaching values down to 2 for nanogaps smaller than 2 nm ^22^. Here, the interfacial liquid is not subject to any strong lateral or vertical confinement. The AFM probe operates in soft tapping thus preventing from any strong vertical confinement of the ions and water molecules^4^. Furthermore, the interfacial liquid is relatively free to laterally move without any major topographical constraints due to nanochannels^22^ or surface defects^23^. Considering the average distance between the ions and the mica surface (~ 0.3 nm for the species considered here^2,24–26^), the average electrostatic energy is ~ - 4 *k_B_T* and ~ - 17 *k_B_T* for monovalent and divalent ions, respectively. As discussed in ref. ^15^, the value for *l_B_* and consequently for *E_el_* are likely an overestimate due to the model assuming ions directly adsorbed to the mica surface. This condition is not necessarily verified here in particular for outer-sphere (OS) complexes (see also Fig. S5 in ESI section 8).

We can now model the total mica-ion electrostatic energy for a pair of neighbouring ions adsorbed to adjacent mica sites (see Fig. S3):

$E_{ind}=2 E_{el} . ($S5)

In addition to *E_ind_*, each cation experiences a direct repulsive interaction with its neighbours and an attractive electrostatic interaction with the binding sites of its neighbours (see Fig. S3). For a pair of ions, this correlation interaction between two adjacent cations is given by

${2 E}_{corr}=\left( +\frac{l_{B}}{d_{2}}-2\frac{l_{B}}{d_{3}} \right)k_{B}T, ($S6)

where *E_corr_* describes the electrostatic correlation contribution per ion. *d_2_* and *d_3_* are taken to be 0.52 nm (corresponding to the mica lattice parameter^4,15^) and 0.60 nm (see Fig. S3). We get that *E_corr_* is ~ - 1 *k_B_T*, and ~ - 4 *k_B_T* for monovalent ions and divalent ions, respectively.

**Fig. S3.** Schematics showing the interactions of metal cations (red spheres) at the mica-water interface. (**a-b**) represent two different situations depending on whether cations are adsorbed adjacent to each other. (**a**) Cations are not adsorbed on adjacent sites and their electrostatic interactions are limited to the negatively charged mica surface binding sites (brown spheres). Electrostatic correlation interactions between cations are prevented from the interposition of hydronium ions (blue spheres). In (**b**), the cations are adsorbed to adjacent sites thus giving rise to attractive correlation interactions. Figure modified from ref. ^15^.

In the case of ionic domains, the total electrostatic energy is given by the interactions of the ions adsorbed adjacent to each other. Thus, the energy can be calculated summing over all the pair interactions:

$E_{pair}=\left( -2\frac{l_{B}}{d_{1}}+\frac{l_{B}}{d_{2}}-2\frac{l_{B}}{d_{3}} \right)k_{B}T= E_{ind}+ 2E_{corr} . ($S7)

For monovalent ions, the electrostatic correlation energy *E_corr_* is approximately equal to thermal fluctuations, thus insufficient to effectively stabilise ionic clusters over the relatively long time scale of several seconds experimentally observed here (see for example the cyan-coloured domain in Fig. 1). Also in the case of Ca^2+^, *E_corr_* is not likely to effectively provide the observed long-lived domains with the required energy to robustly withstand perturbations induced either by thermal fluctuations or by the mechanical/electrostatic interactions due to the scanning probe (~0.5-5 *k_B_T*)^15^.

Notably, *E_corr_* describes the maximum value for the electrostatic correlation interaction in the absence of any other effects due, for example, to specific solvation structure of the ions and their configuration as inner-sphere (IS) or outer-sphere complexes^26^; the pH of the aqueous medium with hydronium ions competing for the mica binding sites^4^; hydration water molecules shielding the cations electric field and volume contraction of the coordinated water^27^. The model, in particular, works under the assumption of ions adsorbed as IS complexes. The approximation may hold true for K^+^ and Rb^+^ which adsorb mainly as IS complexes^24,28^, but Na^+^ and Ca^2+^ tend in reality to adsorb at a greater distance from the mica substrate.

Accurately quantifying the perturbations induced by the scanning probe is also a challenging task^29,30^. Here, we use the results in ref.^15^ to approximate the mechanical and electrostatic perturbations induced by the tip at the interface between mica and ionic aqueous solutions. This approximation relies on the similar characteristics of the AFM cantilevers used here and in ref.^15^, as well as on the identical interface under investigation.

**6. Ion clusters size as a function of charge density**

The greater the charge density, the smaller the attractive water-correlated correlation interactions. This results in an increase in the ion mobility and smaller domains as shown in Fig. S4. Combining the data in Fig. 3b and S4, we calculate the fractal dimension as shown in Fig. 3c. The details of the fractal dimension analysis are discussed in the next ESI section.

**
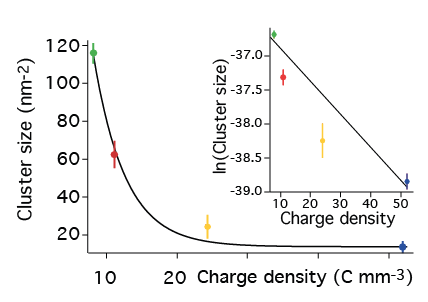
**

**Fig. S4**. The average ion cluster size exponentially decays with the ion charge density.

It is worth mentioning that domain size and fractal dimension can also influence the interaction of the shear flow with clusters as a whole, particularly considering the different area/edge length the drag force is exerted onto. Shear flow may indeed bias the motion of the domains both acting on single ions and on the entire domain. Still, it is reasonable to assume that the shear flow has a limited effect considering the relatively small associated drag force, and hence should not significantly depend on domain size or edge length. This is also consistent with our description of the velocity of the clusters using a simple exponential function which implies a thermally activated process. The model is well-known as suitable for describing individual molecules or ions^4,23^. Here, it also offers a good description of the ionic nanodomains’ dynamics because the charge density of individual ions remains the key parameter that modulates, through water-mediated interactions, the group effects behind domain structure and dynamics. Further experiments at different temperatures are however needed to confirm the extent of the validity for the proposed thermally activated motion of ionic clusters.

Future experiments should also explore the role of salt concentration as a tool to tune structural organisation and dynamics of ionic domains. As shown by recent XRR and molecular dynamics simulations, ion concentration can promote, through water-mediated ion cooperativity effects, over-screening of the charged solid/liquid interface and heterogenous nucleation at the interface^31^. Thus, we may assume that increasing salt concentration would lead to larger and more stable domains up to a point where mesoscale structures start forming.

**7. Fractal dimension analysis**

The fractal dimension of the ion domains was estimated using a custom-made Python algorithm^32^. The algorithm is based on calculating the Minkowski–Bouligand dimension, or box counting dimension^33^. The approach resolves the shape of the object as a function of the boxes needed to cover the entire image. Given *N(ε)* boxes with side length *ε,* the fractal dimension is given by

$dim=\lim_{\epsilon\to o} \frac{\log N(\epsilon)}{\log\left( \frac{1}{\epsilon} \right)}$ . (S6)

The software was tested using the coastline of Great Britain. Our routine returned a fractal dimension of 1.26 in excellent agreement with the literature value of 1.25 ^34^.

**8. Pictorial representation of inner-sphere and outer-sphere complexes**

At the water/mica interface, the two main hydration configurations adopted by adsorbed metal cations are the so-called inner-sphere (IS) and outer-sphere (OS) complexes, with several potential pathways for exchange between the hydration state and the solution species^24,35^. This is pictorially represented as a cartoon in Fig. S5 below.

**Fig. S5**. Adsorption of metal cations at the water/mica interface as partially dehydrated inner-sphere (IS) and more hydrated outer-sphere (OS) complexes. IS complexes are directly adsorbed to the mica surface, whereas for OS complexes a water molecule/hydroxyl anion is present between the ion and the substrate.

**9. Stability over time of ionic domains**

As shown in Fig. 1, ionic domains may undergo restructuring as a consequence of their interactions with neighbouring clusters. The time evolution of the domains is influenced by their size, itself controlled by ion-ion correlation effects that depend on the ions’ charge density *ρ*. As a result, changes in domain size and in number of ions per domain linearly depend on *ρ*, with Rb^+^ and K^+^ forming larger and more stable domains (see Fig. S6). This is because increasing *ρ* tends to disfavour attractive correlation interactions between ions resulting in relatively highly mobile clusters subject to more pronounced reshaping.

**Fig. S6**. Time evolution of ionic domains. For each type of ion, the change over time in cluster size and in the number of ions per cluster are measured over a period of more than 6 seconds (4 images) by comparing consecutive images. The results are then averaged and normalised by the average cluster size (number of ions, respectively). The domain evolution and velocity are such that domains do not survive for more than 10-12 seconds. When a smaller domain merges with a larger domain, the travel distance and the velocity are automatically computed by the computer algorithm with respect to the latter. This approach avoids arbitrary and potentially misleading identification of the original domains.

**10. Cleaning procedure and extended methods**

AFM measurements are sensitive to contaminants which may lead to anomalous and inconsistent data. Extensive cleaning procedures were implemented so as to ensure full reliability of the results^4,21,23,36^.

Ionic aqueous solutions were prepared and stored in borosilicate bottles (Pyrex, Corning, NY, USA). Borosilicate containers underwent the following protocol before use. First, they were washed with tap water and soap, then rinsed with tap water 20 times, followed by ultra-pure water (18.2 MΩ, Merck Millipore, Billerica, MA, USA), > 98% pure propan-2-ol (Merck Millipore, Billerica, MA, USA), and finally ultra-pure water. The propan-2-ol was used without any further purification. The glass containers were extensively flushed with nitrogen so as to remove any remaining water.

AFM cantilevers were cleaned in a bath of ultrapure water, followed by propan-2-ol, and finally ultrapure water again. Each step lasted for at least 20 minutes. The AFM stage was also subject to an extensive cleaning procedure. It was firstly cleaned with ultra-pure water, then pure propan-2-ol, and finally with ultra-pure water.

**Removal of the K+ ions initially present at the surface of the muscovite mica**

K+ ions initially present at the surface of the muscovite mica may interfere with the measurements. When a mica surface is freshly cleaved in air, K+ ions cover half of the ditrigonal cavities. These ions come from the cleavable layers of the crystal bulk and ensure electrostatic neutrality^4^. Following the procedure detailed in ref. ^4^, we ensured that that the final concentration of these ions was at least four orders of magnitude smaller than the concentration of the desired ions.

In brief, the mica surface is a 12 mm diameter disc. The mica disc area is ~ 4.5×10-4 m2. The area of a hexagonal lattice site is ~ 2.2×10-19 m2, thus a total of ~ 1×1015 K+ ions are estimated to be exposed on a cleaved surface. This corresponds to 2 nanomoles of K+. The mica surface was rinsed 20 times with 100 μl of ultrapure water, followed by rinsing 40 times with 100 μl of the desired ionic aqueous solution. Considering the total volume of the rinsing liquid, the final K+ concentration on the surface is ~ 0.3 μM. The calculated concentration, however, overestimates the actual number of K+ ions on the surface. This is because the calculation assumed single rinsing. Sequential rinsing is, however, more efficient in diluting K+ cations in comparison to single rinsing^4^.

**11. Supplementary References**

1. Trewby, W., Faraudo, J. & Voïtchovsky, K. Long-lived ionic nano-domains can modulate the stiffness of soft interfaces. *Nanoscale* **11**, 4376–4384 (2019).

2. Adapa, S., Swamy, D. R., Kancharla, S., Pradhan, S. & Malani, A. Role of mono- and divalent surface cations on the structure and adsorption behavior of water on mica surface. *Langmuir* **34**, 14472–14488 (2018).

3. Mate, M. & Carpick, R.W. Tribology on the small scale: a modern textbook on friction, lubrication and wear (Oxford University Press, 2019).

4. Cafolla, C. & Voïtchovsky, K. Lubricating properties of single metal ions at interfaces. *Nanoscale* **10**, 11831-11840 (2018).

5. Aketagawa, M. & Takada, K. Correction of distorted STM image by using a regular crystalline lattice and 2D FFT. *Nanotechnology*, **6**, 105 (1995).

6. Ophus, C., Ciston, J. & Nelson, C. T. Correcting nonlinear drift distortion of scanning probe and scanning transmission electron microscopies from image pairs with orthogonal scan directions. *Ultramicroscopy* **162**, 1–9 (2016).

7. Salmons, B. S., Katz, D. R. & Trawick, M. L. Correction of distortion due to thermal drift in scanning probe microscopy. *Ultramicroscopy* **110**, 339–349 (2010).

8. Lapshin, R. V. Automatic drift elimination in probe microscope images based on techniques of counter-scanning and topography feature recognition. *Meas. Sci. Technol.* **18**, 907–927 (2007).

9. Bowen, W. R. & Doneva, T. A. Artefacts in AFM studies of membranes: Correcting pore images using fast fourier transform filtering. *J. Memb. Sci.* **171**, 141–147 (2000).

10. Nussbaumer, H. J. Fast Fourier transform and convolution algorithms. 80–111 (Springer, 1981).

11. Zhang, J. *et al.* Unconventional atomic structure of graphene sheets on solid substrates. *Small* **15**, 1902637 (2019).

12. Fukuma, T., Ueda, Y., Yoshioka, S. & Asakawa, H. Atomic-scale distribution of water molecules at the mica-water interface visualized by three-dimensional scanning force microscopy. *Phys. Rev. Lett.* **104**, 016101 (2010).

13. Kestin, J., Sokolov, M. & Wakeham, W. A. Viscosity of liquid water in the range -8 °C to 150 °C. *J. Phys. Chem. Ref. Data* **71**, 941–948 (1978).

14. Tansel, B. *et al.* Significance of hydrated radius and hydration shells on ionic permeability during nanofiltration in dead end and cross flow modes. *Sep. Purif. Technol.* **51**, 40–47 (2006).

15. Ricci, M., Spijker, P. & Voïtchovsky, K. Water-induced correlation between single ions imaged at the solid-liquid interface. *Nat. Commun.* **5**, 4400 (2014).

16. Naji, A. & Netz, R. R. Attraction of like-charged macroions in the strong-coupling limit. *Eur. Phys. J. E 2004 131* **13**, 43–59 (2004).

17. Khan, M. O. & Jonsson, B. Electrostatic correlations fold DNA. *Biopolymers* **49,** 121-125 (1999).

18. Janeček, J. & Netz, R. R. Effective screening length and quasiuniversality for the restricted primitive model of an electrolyte solution. *J. Chem. Phys.* **130**, 074502 (2009).

19. Low, C. G., Zhang, Q., Hao, Y. & Ruoff, R. S. Graphene field effect transistors with mica as gate dielectric layers. *Small* **10**, 4213–4218 (2014).

20. Low, C. G. & Zhang, Q. Ultra-thin and flat mica as gate dielectric layers. *Small* **8**, 2178–2183 (2012).

21. Cafolla, C. & Voïtchovsky, K. Impact of water on the lubricating properties of hexadecane at the nanoscale. Nanoscale **12**, 14504-14513 (2020)

22. Fumagalli, L. *et al.* Anomalously low dielectric constant of confined water. *Science* **360**, 1339–1342 (2018).

23. Cafolla, C., Foster, W. & Voïtchovsky, K. Lubricated friction around nanodefects. *Sci. Adv.* **6**, eaaz3673 (2020).

24. Lee, S. S., Fenter, P., Nagy, K. L. & Sturchio, N. C. Monovalent ion adsorption at the muscovite (001)−solution interface: relationships among ion coverage and speciation, interfacial water structure, and substrate relaxation. *Langmuir* **28**, 8637-8650 (2012).

25. Park, C., Fenter, P. A., Nagy, K. L. & Sturchio, N. C. Hydration and distribution of ions at the mica-water interface. *Phys. Rev. Lett.* **97**, 016101 (2006).

26. Schlegel, M. L. *et al.* Cation sorption on the muscovite (001) surface in chloride solutions using high-resolution X-ray reflectivity. *Geochim. Cosmochim. Acta* **70**, 3549–3565 (2006).

27. Yang, Z. H. The size and structure of selected hydrated ions and implications for ion channel selectivity. *RSC Adv.* **5**, 1213–1219 (2015).

28. Lee, S. S., Fenter, P., Nagy, K. L. & Sturchio, N. C. Changes in adsorption free energy and speciation during competitive adsorption between monovalent cations at the muscovite (001)-water interface. *Geochim. Cosmochim. Acta* **123**, 416–426 (2013).

29. Anczykowski, B., Gotsmann, B., Fuchs, H., Cleveland, J. P. & Elings, V. B. How to measure energy dissipation in dynamic mode atomic force microscopy. *Appl. Surf. Sci.* **140**, 376–382 (1999).

30. Katan, A. J., Van Es, M. H. & Oosterkamp, T. H. Quantitative force versus distance measurements in amplitude modulation AFM: a novel force inversion technique. *Nanotechnology* **20**, 165703 (2009).

31. Lee, S. S., Koishi, A., Bourg, I. C. & Fenter, P. Ion correlations drive charge overscreening and heterogeneous nucleation at solid–aqueous electrolyte interfaces. *Proc. Natl. Acad. Sci.* **118**, e2105154118 (2021).

32. Theiler, J. Estimating fractal dimension. *J. Opt. Soc. Am. A* **7**, 1055-1073 (1990).

33. Moreira, J. G., Da Silva, J. K. L. & Kamphorst, S. O. On the fractal dimension of self-affine profiles. *J. Phys. A. Math. Gen.* **27**, 8079–8089 (1994).

34. Fractal Foundation. *Fractal Dimension* http://fractalfoundation.org/OFC/OFC-10-4.html (2021)

35. Lee, S. S., Fenter, P., Nagy, K. L. & Sturchio, N. C. Real-time observation of cation exchange kinetics and dynamics at the muscovite-water interface. *Nat. Commun.* **8**, 15826 (2017).

36. Miller, E. J. *et al.* Sub-nanometer Resolution Imaging with Amplitude-modulation Atomic Force Microscopy in Liquid. *J. Vis. Exp.* **118**, 54924 (2016).
